# The correlation between fibroblast growth factor-23 and ESRD patients with hearing impairment

**DOI:** 10.7717/peerj.12295

**Published:** 2021-10-13

**Authors:** Jingwen Nie, Qing Li, Min Guo, Jiaqing Li, Jiahui Yang, Qing Chang, Yaping Cai

**Affiliations:** 1 Department of Nephrology, The First Affiliated Hospital of Kunming Medical University, Kunming, China; 2 Department of Otolaryngology, The First Affiliated Hospital of Kunming Medical University, Kunming, China

**Keywords:** Hearing impairment, Fibroblast growth factor-23, End-stage renal disease, Chronic kidney disease

## Abstract

**Background:**

End-stage renal disease (ESRD) patients often experience hearing impairment, resulting in a high rate of disability and a decline in their quality of life. Fibroblast growth factor-23 (FGF23) is a diagnostic biomarker for chronic kidney disease (CKD) and a pathogenic contributor to CKD progression. However, the correlation between FGF23 level and CKD patients with hearing impairment remains elusive. This study aimed to investigate the relationship between the FGF23 and ESRD accompanied with hearing impairment.

**Methods:**

A total of 144 ESRD patients, who were admitted to the First Affiliated Hospital of Kunming Medical University from November to December 2020, were enrolled in this study. Firstly, 144 ESRD patients underwent pure-tone audiometry (PTA). Secondly, it was attempted to randomly select 20 ESRD patients with normal hearing, and 20 ESRD patients with hearing impairment (match ratio, 1:1). Age- and gender-matched healthy people (*n* = 20) were also recruited as controls group. The expression levels of FGF23 was detected by enzyme-linked immunosorbent assay (ELISA).

**Results:**

The results of pure-tone audiometry showed that the prevalence of hearing impairment in ESRD patients was 80.5%. Male ESRD patients were more likely to develop hearing impairment compared to female patients. The incidence rate of hearing impairment at a high frequency was significantly higher than that at a low frequency (*P* < 0.01). The serum levels of FGF23, phosphorus, and parathyroid hormone (PTH) in ESRD patients with hearing impairment significantly increased compared with those with normal hearing and healthy controls.

**Conclusion:**

ESRD patients had a higher risk of hearing loss, especially high-frequency hearing impairment. As FGF23 level increased, the risk of hearing loss was also elevated. The hearing impairment in ESRD patients was associated with the degree of kidney injury, and serum FGF23 level.

## Introduction

With the aging of population, the prevalence of chronic kidney disease (CKD) has risen to 14.3% worldwide, accounting for more than 850 million patients. CKD has become a major public health problem (Mehta et al., 2015). With the progression of the disease, CKD may develop into end-stage renal disease (ESRD). ESRD patients mainly suffer from hearing impairment (HI) (Lee et al., 2019). The incidence of HI, especially sensorineural hearing loss (SNHL), is as high as 36–77%, suggesting that chronic renal failure may damage the auditory nervous system (Gupta et al., 2020; Seo et al., 2015; Yueniwati & Apprianisa, 2019). HI is the fifth leading cause of disability worldwide (Machado-Fragua et al., 2021). HI is one of the risk factors for cognitive decline, which is independently associated with dementia (Hardy et al., 2016; Heywood et al., 2017; Saji et al., 2021).

In 2000, fibroblast growth factor-23 (FGF23) was firstly discovered and named by Yamashita, Yoshioka & Itoh (2000). It is a regulatory factor that is produced and secreted by osteocytes and osteoblasts, and belongs to the polypeptide hormone of the endocrine FGFs. Under normal physiological conditions, FGF23 can promote the excretion of phosphorus in the blood and urine, and inhibit the conversion of 25-OH-VitD_3_ to 1, 25(OH)_2_VitD_3_, leading to a negative phosphorus balance. In kidney diseases, the compensatory increase of FGF23 inhibits the synthesis of 1,25(OH)_2_VitD_3_, thereby maintaining the normal serum phosphorus concentration. FGF23 may play a pivotal role in mineral ion disorders and bone metabolism.

Klotho was discovered incidentally in 1997 by Kuro-o et al. (1997). It can exert the effects of anti-aging, enhancing cognitive ability, and regulating mineral metabolism (Chen et al., 2020). The klotho gene is expressed in the stria vascularis and spiral ligaments of the inner ear, as well as in the distal convoluted tubule of the renal (Ullah & Sun, 2019). Klotho may play a role in regulation of multiple calcium and potassium ion channels, in addition to various cellular carriers (*e.g.*, Na(+)-coupled cotransporters).

FGF23/Klotho axis can regulate phosphate and vitamin D metabolism in ESRD patients (Ide et al., 2018; Kinoshita & Kawai, 2016). The underlying mechanism of renal and inner ear damage may share some similarities, but the exact mechanism remains elusive. The present research aimed to assess the correlation between FGF23 and ESRD accompanied with HI.

## Materials and Methods

### Study population and sampling technique

**Figure 1:**
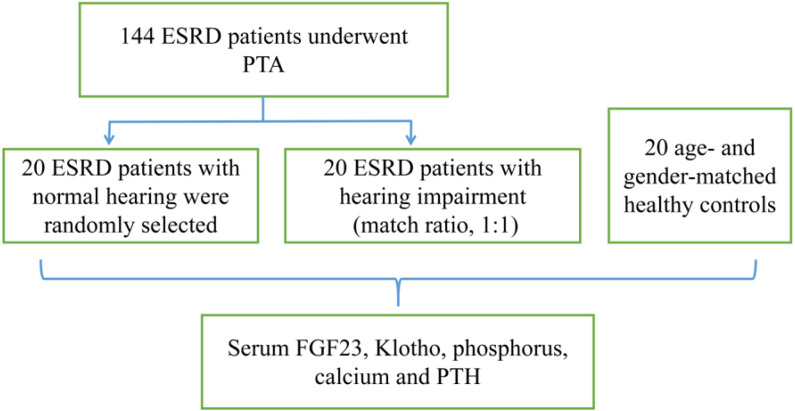
Flowchart of the trial selection.

A total of 144 ESRD patients, who were admitted to the First Affiliated Hospital of Kunming Medical University (Kunming, China) from November to December 2020, were enrolled in this research. Firstly, 144 ESRD patients underwent pure-tone audiometry (PTA). Secondly, it was attempted to randomly select 20 ESRD patients with normal hearing, and 20 ESRD patients with HI (match ratio, 1:1), as well as recruiting 20 age- and gender-matched healthy controls (control group) (Fig. 1).

### Data collection

#### Questionnaire design

A structured questionnaire was designed to collect basic socio-demographic data related to the patients’ age, gender, educational level, marital status, and complications (*e.g.*, radiation protopathy).

#### Laboratory data

The laboratory test results included the levels of hemoglobin (Hb), blood urea nitrogen (BUN), serum creatinine (Cre), potassium, calcium, parathyroid hormone (PTH), and phosphorus.

#### PTA

A Conera pure tone audiometer (MADSEN Co., Ltd., Copenhagen, Denmark) was used to perform PTA in both ears at the frequencies of 250, 500, 1000, 2000, 4000, and 8000 Hz, and the mean value was calculated.

#### The serum levels of FGF23 and Klotho

Eight-h fasting venous blood samples (5 mL) were collected from all subjects before 8:30 am, and the supernatant was extracted through centrifugation for 10 min at 3,000 rpm/min. The serum levels of FGF23 and Klotho were detected by using commercial enzyme-linked immunosorbent assay (ELISA) kits (Elabscience Co. Ltd., Wuhan, China) according to the manufacturer’s instructions.

### Inclusion and exclusion criteria

The inclusion criteria were as follows: patients’ age ≥ 18 years old; glomerular filtration rate (GFR) < 15 mL/min⋅1.73 m^2^; patients with consciousness and communication ability; receiving hemodialysis three times/week over a period of longer than 3 months.

HI was measured by PTA, and was defined as PTA ≧ 25 dB hearing level (dB HL) in either ear at any frequency. Low/intermediate frequency HI was defined as the average hearing threshold ≧ 25 dB HL in one ear at the frequencies of 250, 500, 1000 and 2000 Hz. High frequency HI was defined as the average hearing threshold ≧ 25 dB HL in one ear at the frequencies of 4000 and 8000 Hz.

The exclusion criteria were as follows: recent major surgery, hemodialysis period, long-term noise exposure, malignant tumors, history of acute otitis media, family history of deafness, history of administration of ototoxic drugs, and unwillingness to cooperation.

### Ethical approval

The study was approved by the Ethics Committee of Kunming Medical University, China, and it was conducted in accordance with the Declaration of Helsinki. All participants read and signed the written informed consent form.

#### Statistical analysis

EpiData 3.1 software was used to establish the database. Chi-square test was used for analysis of categorical variables. The Student’s *t*-test was employed to evaluate differences between continuous variables. The influencing factors of HI were analyzed by the multivariate logistic regression analysis. A *P*-value < 0.05 was considered statistically significant. In addition, all statistical analyses were performed using SPSS 17.0 software (IBM, Armonk, NY, USA).

## Results

### General characteristics and hearing screening of ESRD patients

The general characteristics of the ESRD patients were presented in Table 1. In the present study, 144 ESRD patients (288 ears) were screened, of whom, 116 cases had HI. Male patients had a higher incidence of HI than female ones (*P* < 0.01). Both ears or a single ear suffered HI, mainly with damage to both ears (20/96 cases). Besides, 46 ESRD cases (66 ears) had HI, while no clinical manifestations could be observed, suggesting subclinical HI.

**Table 1:** The general characteristics of the ESRD patients.

Characteristic	Male	Female	ALL
Age (y), mean (SD)	42.8 (11.5)^**^	52.2 (15)	47.8 (14.2)
N (%)	68 (47.2)	76 (52.8)	144 (100.0)
BMI (Kg/m^2^), mean (SD)	23.2 (3.8)^**^	21.5 (3.9)	22.3 (3.9)
History of hypertension (%)	68 (47.2)	68 (47.2)	136 (94.4)
History of diabetes mellitus (%)	8 (5.6)	12 (8.3)	20 (13.9)
HB (g/L), mean (SD)	114.3 (21.3)	108.6 (19.6)	111.3 (20.5)
Duration (month), mean (SD)	40.9 (38.4)	43.7 (32.4)	42.4 (35.3)
CKD etiology n (%)			
Chronic glomerulonephritis	56 (38.9)	51 (35.4)	107 (74.3)
Diabetic nephropathy	8 (5.6)	12 (8.3)	20 (13.9)
Hypertensive nephropathy	0 (0.0)	9 (6.3)	9 (6.3)
Other	4 (2.8)	4 (2.8)	8 (5.6)
Creatinine (umol/l), mean (SD)	1073.6 (354.0)^*^	964.0 (218.1)	1015.8 (294.4)
BUN (mmol/l), mean (SD)	24.6 (6.4)	26.7 (7.5)	25.7 (7.1)
Potassium (mmol/l), mean (SD)	5.2 (0.7)	5.2 (0.8)	5.2 (0.7)
Calcium (mmol/l), mean (SD)	2.3 (0.2)	2.4 (0.2)	2.4 (0.2)
Phosphorus (mmol/l), mean (SD)	2.2 (0.5)	2.0 (0.4)	2.1 (0.4)
PTH (pg/mL) , mean (SD)	579.7 (571.5)	481.0 (340.2)	527.6 (464.9)
Hearing impairment N (%)	64 (44.4)^**^	52 (36.1)	116 (80.5)
Both ears damage N (%)	56 (38.9)^**^	40 (27.8)	96 (66.7)
Single ear damage N (%)	8 (5.6)	12 (8.3)	20 (13.9)

**Notes.**

* *P* < 0.05.

** *P* < 0.01.

### PTA

The results of PTA were presented in Table 2. The rate of HI at a high frequency was significantly higher than that at a low frequency.

**Table 2:** The results of PTA of ESRD patients.

	Low/intermediate-frequency (Hz)			High-frequency (Hz)		
	250	500	1000	2000	4000	8000
Left ear (dB)	20.7 ± 10.6	19.9 ± 8.8	21.7 ± 10.4	23.2 ± 14.7	35.0 ± 19.5	39.9 ± 23.7
Right ear (dB)	20.6 ± 8.2	18.6 ± 7.8	18.5 ± 9.6	20.6 ± 13.1	31.5 ± 16.7	38.5 ± 22.6
Hearing impairment N (%)	44 (30.6)	32 (22.2)	52 (36.1)	48 (33.3)	92 (63.9)	108 (75.0)
Total N (%)	176 (30.6)				200 (92.4)	

#### Comparing the serum levels of FGF23 and Klotho among the three groups

It was attempted to randomly select 20 ESRD patients with normal hearing, and 20 ESRD patients with HI (match ratio, 1:1), as well as recruiting 20 age- and gender-matched healthy controls (control group). The comparison of the serum levels of FGF23, Klotho, PTH, calcium, and phosphorus among the three groups were shown in Table 3.

**Table 3:** Comparing the serum level of biochemical indicators among the three groups (X ± S).

	ESRD+HI group	ESRD+NH group	Control group
Age (y)	33.8 ± 3.9	33.8 ± 4.1	34.6 ± 5.9
BMI (kg/m^2^)	22.2 ± 4.8	22.6 ± 4.9	22.7 ± 3.6
FGF23 (ng/L)	699.8 ± 334.9 ^**^	607.3 ± 323.6	61.2 ± 17.9
Klotho (pg/ml)	16.2 ± 2.3	16.7 ± 2.8	16.4 ± 3.8
PTH (pg/mL)	1083.1 ± 783.5 ^**^	613.9 ± 395.6	30.8 ± 14.5
Calcium (mmol/l)	2.3 ± 0.2	2.5 ± 0.2	2.2 ± 0.1
Phosphorus (mmol/l)	2.1 ± 0.2 ^**^	2.2 ± 0.4	1.17 ± 0.15
Potassium (mmol/l)	5.4 ± 0.6 ^**^	4.9 ± 0.7	3.7 ± 0.2
Cre (umol/l)	1152.6 ± 307.6 ^**^	1012.7 ± 194.7	77.0 ± 18.7
BUN (mmoll)	25.1 ± 3.2 ^**^	24.3 ± 6.5	6.0 ± 2.7

**Notes.**

HI Hearing impairment NH Normal hearing

* *P* < 0.05.

** *P* < 0.01.

The results showed that the serum levels of FGF23, PTH, potassium and phosphorus in ESRD + HI group were higher than those in NH group and control group (*P* < 0.01). However, there was no significant difference in serum levels of Klotho and calcium among the three groups.

#### Influencing factors of HI in ESRD patients

We defined the hearing impairment as a dependent variable (0 = none, 1 = yes), and the predictive factors used in univariate analysis as independent variables. Comparing the 3 groups, there were significant differences in 12 variables including age (0 < 50y, 1 ≧ 50y), gender (0 = male, 1 = female), smoking (0 = none, 1 = yes), hypertension (0 = none, 1 = yes), diabetes (0 = none, 1 = yes), CKD etiology (1 = chronic glomerulonephritis, 2 = diabetic nephropathy, 3 = hypertensive nephropathy, 4 = other), anemia (0 = none, 1 = yes), FGF23 (1< 800 ng/L=, 2 ≧ 800 ng/L), calcium (0 < 2.3 mmol/l, 1 ≧ 2.3 mmol/l), phosphorus (0 < 2 mmol/l, 1 ≧ 2 mmol/l), potassium (0 < 5.2 mmol/l, 1 ≧ 5.2 mmol/l), duration of CKD (1 < 30 months, 2 ≥ 30 months). Therefore, these 12 factors were considered as possible influencing factors, and multivariate logistic regression was performed. The results (Table 4) showed that age, gender, smoking and FGF23 were independent risk factors for HI (*P* < 0.05).

**Table 4:** Multivariate regression analysis of influencing factors of HI in CKD.

Variables	B	S.E.	*P*	OR	95% CI
Gender	−2.531	0.684	<0.001	0.08	(0.021, 0.304)
Age	2.24	0.574	<0.001	9.392	(3.048, 28.939)
Smoking	1.747	0.837	0.037	5.739	(1.112, 29.614)
FGF23	1.135	0.49	0.021	3.111	(1.19, 8.132)

## Discussion

According to the report released by the World Health Organization, approximately 466 million people (or 6.1% of the global population) were living with HI in 2018 (Puga et al., 2018). Sudden SNHL in ESRD patients is a marker of pathogenic progression in the mortality and atherosclerotic events (Boateng et al., 2019; Chou et al., 2018; Saeed et al., 2018). The risk of developing sudden SNHL in ESRD patients was found 2.17 times higher than that in normal population (Wang et al., 2017). ESRD is an independent risk factor for HI. A number of scholars have pointed out that with the recovery of kidney function in patients who received renal transplantation, HI is cured or alleviated (Bains et al., 2007). Compared with the study by Jishana et al. (2015), this study revealed that the incidence of HI in ESRD patients was higher and the majority of ESRD patients had SNHL. The difference may be due the evaluation criteria of hearing damage in the two studies. Jishana et al. used pure-tone-average as the standard of hearing loss. Our study defined hearing impairment as PTA ≧ 25 dB hearing level (dB HL) in either ear at any frequency. Moreover, there were also differences in patients’ age, gender and noise exposure history between the two studies. People’s daily communication is always carried out at the low and medium frequencies (500, l000, and 2000 Hz). However, HI often appears at high frequencies (4000 and 8000 Hz), and patients often have no clinical symptoms at the beginning (Kuang et al., 2021, unless hearing loss could reach low frequencies (500, l000, 2000 Hz), indicating that the HI is an insidious process (Moore, 2016). As a result, HI is prevalent, and ESRD patients should undergo hearing test as early as possible, and receive early intervention if required. Thus, systemic metabolic disorders caused by the ESRD might lead to impairment of inner ear and HI.

The progression of CKD is associated with oxidative stress and inflammatory response, which are responsible for the manifestation of numerous comorbidities such as chronic heart failure, malnutrition, calciphylaxis, artery calcification, anemia, as well as mineral and bone disorders (Nakanishi et al., 2019). Numerous factors can contribute to cochlear dysfunction in CKD patients. The kidney and the cochlea show remarkable similarities, such as tubular organization, glomerular membranous structures and the central role of ciliated epithelial cells (Gratton et al., 2005). The oxygen consumption of these two organs is remarkable. Consequently, the inner ear’s stria vascularis and the glomerular basement membrane have the same antigenicity, and both are associated with immunities (Manou et al., 2020).

The present study showed that age, gender, smoking and FGF23 were independent influencing factors for HI. ESRD patients with higher FGF23 levels had a 3.11 times higher risk of HI than those with lower FGF23 levels. The increased serum FGF23 level in ESRD patients was associated with hyperphosphatemia and hyperparathyroidism. Moreover, there was a correlation between serum FGF23 level and HI. A number of researches demonstrated that FGF23 can regulate a variety of signaling pathways in human body, such as mineral metabolism, insulin resistance, energy balance, premature aging, etc. (Lacroix & Urena-Torres, 2020; Milovanova et al., 2018). Combination of Klotho with fibroblast growth factor receptor 1 (FGFR1) can form a transmembrane co-receptor of FGF23 to regulate FGF23/FGFR1/Klotho signal transduction.

The present study showed that serum Klotho values did not differ between ESRD patients and healthy controls. The reasons might be as follows. Firstly, Klotho protein was highly expressed in kidney. Renal Klotho mRNA and protein were decreased in db/db mice (Zh et al., 2011; Asai et al., 2012). In CKD patients, urine Klotho was even more sensitive and the magnitude of its reduction was correlated with the severity of the diminution in eGFR (Hu et al., 2011). Moreover, a stepwise multiple regression analysis considered eGFR to be an independent factor related only to urine Klotho but not to serum Klotho, suggesting that urine Klotho testing might be more credible than serum Klotho test (Akimoto et al., 2012). Therefore, the present study only examined serum Klotho. Secondly, the majority of the current reports were based on mouse model. Clinical data related to Klotho and FGF23 came from small size observational studies, and larger cohorts were needed to further confirm the results.

With the decline of renal function and metabolites, *e.g.*, creatinine, blood urea nitrogen, phosphorus, the FGF23/Klotho signaling pathway can be activated (Gabr, Kotait & Okda, 2019). High levels of FGF23 and hyperphosphatemia may cause damage to cochlea’s capillary, leading to cochlear sclerosis, and may also affect the enzymatic activity and ion exchange in the inner ear and reduce the excitability of cochlear neurons (Khodeir, Okda & Abdalal, 2019).

Lysaght et al. (2014) reported that FGF23 null mice developed mixed hearing loss. Their findings demonstrated that the lack of FGF23 caused extensive sensory impairment in mice. FGF23 null mice exhibited severe hypercalcemia, hyperphosphatemia and hypervitaminosis D, leading to demineralization of the cochlea. The present study revealed that elevated serum FGF23 level in ESRD patients was associated with hyperphosphatemia, hyperparathyroidism and hypovitaminosis D, resulting in demyelination and demineralization of the cochlea. Moreover, hyperphosphatemia and hyperparathyroidism could change the fluid and electrolyte composition of endolymph. We assumed that the effect of FGF23 on the auditory systems might present a “U-shaped” curve. Too high or too low levels of FGF23 might affect auditory function.

Since serum FGF23 level was increased in ESRD patients, we speculated that FGF23 inhibitor could reduce the level of phosphorus and parathyroid hormone, thereby alleviating the demineralization and demyelination of the cochlea. Of course, further experiments were needed to verify this hypothesis. This proximity is clinically relevant since lesions to this nerve may typically produce symptoms in both the glomerular and auditory components (Barilyak et al., 2018; Fakhar & Rashid, 2017; Quarles, 2019).

In summary, our findings revealed that excessive activation of FGF23 is a risk factor for the progression of HI in ESRD patients. With monitoring the serum FGF23 level as a potential biomarker, HI in ESRD patients can be early diagnosed and effectively treated. In the future, FGF23 inhibitor may be utilized as a potential therapeutic target for HI in ESRD patients. However, the results did not detect the expression level of FGF23 in cochlea and kidney tissues. Thus, a mouse model with CKD and HI will be established in the next research. We plan to establish FGF23-overexpression and FGF23-knockdown mouse models. The auditory brain response will be tested by Tucker Davis Technologies. Furthermore, the expression level of FGF23 in mouse’s cochlea and kidney tissues will be examined.

## Limitations

The present study had some limitations. First, the sample was small, hindering full reflection of the characteristics of the whole population. Second, the influence of dialysis on hearing remained elusive. Third, the present study only examined serum Klotho, whereas other important factors that might affect hearing, such as kidney Klotho mRNA, urine Klotho and thyroid function, were not investigated. Furthermore, the regulatory mechanism in pathophysiology of the cochlea and kidney were not fully understood. Thus, further study is needed to verify our findings.

## Conclusions

It was revealed that the incidence of HI in ESRD patients is remarkable, which is mainly manifested as high-frequency HI. Besides, overexpression of the FGF23 may be an important factor, leading to HI in ESRD patients.

## Supplemental Information

10.7717/peerj.12295/supp-1 Supplemental Information 1 Raw data

10.7717/peerj.12295/supp-2 Supplemental Information 2 Questionnaire (English

10.7717/peerj.12295/supp-3 Supplemental Information 3 Questionnaire (Chinese)

## Abbreviations

HI: Hearing impairment
ESRD: End-stage Renal Disease
PTA: Pure tone audiometry
SNHL: Sensorineural hearing loss
CKD: Chronic kidney disease
FGF23: Fibroblast growth factor- 23
ELISA: Enzyme-linked immunosorbent assay
HL: Hearing level
MHD: Maintenance hemodialysis

## Additional Information and Declarations

### Data Availability

The following information was supplied regarding data availability: The raw measurements are available in a Supplementary File.
