# Supplementary material for: The correlation between fibroblast growth factor-23 and ESRD patients with hearing impairment
Source: PeerJ. 2021 Oct 13;9:e12295. doi: 10.7717/peerj.12295 (PMC8520394; doi:10.7717/peerj.12295)
Supplement: Supplemental Information 2 [file peerj-09-12295-s002.pdf]

No：□□□□□□□

Questionnaire about chronic kidney disease with hearing loss

Instruction

A. This questionnaire is used to find out about your health. This information will help to document how you feel and how competent you are in your daily activities.

B. This questionnaire includes a wide range of questions about your health and life.

C. Please circle the appropriate number or fill the answers as required.

D. Some questions about the impact of kidney disease on your life, and some questions about the limitation of kidney disease on your life. Please answer each question carefully. If you are not sure how to answer a question, try to give an answer that is as close as to your thoughts. This will enable us to have a better understanding of the different types of respondents with kidney disease.

Thank you for the participating.

I. Basic information

A1. Name: _________, age _________, gender: ① male ② female

A2. Education _______

A3. Nationality: ① Han ② Minorities _______

A4. The number of family members _____

A5. Occupation ① unemployed ② farmer ③ worker ④ self-employed
⑤ staff member ⑥ retirees ⑦ cadres or teachers ⑧ others _______

A6. Marital status: ① unmarried ② married or cohabiting ③ divorced
④ widowed⑤ other ______

A7. Medical insurance: ① self-paid ② basic medical insurance for urban workers
③ basic medical insurance for urban residents ④ free medical treatment
⑤ commercial medical insurance ⑥ new rural cooperative medical care
⑦ others ________

A8. The medical institution?______________________ (name of the hospital)

A9. The monthly income before the illness ￥________,
the current monthly income￥________

A10. Before the illness, the monthly family income was ￥ ________,
the current monthly family income ￥________

A11. History of smoking? ① None (jump to A14) ② Yes

A12. Take ________ cigarettes/day

A13. Smoking ________ years

A14. Do you drink alcohol? ① None (jump to A147) ② Yes

A15. The amount of alcohol ________ml a day

A16.Have you been taking medicine long?

① None (jump to A14) ② type of medicine ________

II. Disease management

B1. Your diagnosis is ________________________.(Hospital records)

B1-1 Date of first diagnosis: __ ____ (month)

B1-2.The stage of your kidney disease _____

(1) CKD- I (2) CKD- II (3) CKD-III (4) CKD-IV (5) CKD-V

B2. The primary disease of your kidney disease _____

(1) Chronic glomerulonephritis (nephrotic syndrome) (2) Diabetic nephropathy

(3) Hypertensive nephropathy (4) Obstructive nephropathy (kidney stones, etc.)

(5) Gouty nephropathy (6) Polycystic kidney (7) Others

B3. Do you have comorbidities?

(1) hypertension (2) itchy skin (3) anemia (4) bone pain (renal bone disease)

(5) limping (6) cramps in legs and feet (7) others (8) no

B4. How do you treat your kidney disease?

(1) hemodialysis (2) peritoneal dialysis (3) renal transplantation

(4) no treatment (5) other _________

B4-1. Date of starting treatment: ___________

B5. Do you feel hearing loss? (1) Yes (2) No (Please skip to B9)

B6. If you have experienced hearing loss, how long does it last?

(1) Days (2) Half a month to one month (3) 1-6 months

(4)7-12 months (5) More than 1 year

B7. Unilateral or bilateral hearing loss?

(1) Unilateral hearing loss. (2) Bilateral hearing loss

B8. What is the impact of hearing loss on work life (VAS visual analog score 0-10)

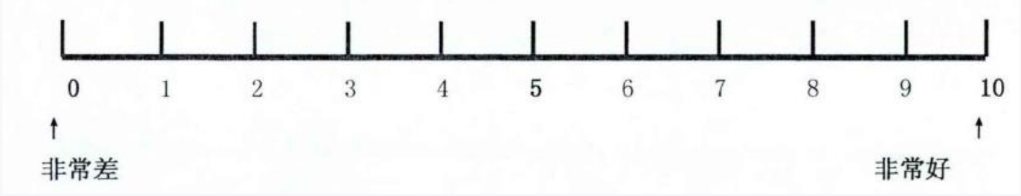

B9. Do you have tinnitus? (1) Yes (2) No (Please skip to B14)

B10. If you have tinnitus, how long does it last?

(1) Days (2) Half a month to one month (3) 1-6 months

(4)7-12 months (5) More than 1 year

B11. Is unilateral or bilateral tinnitus? (1) Unilateral tinnitus (2) Bilateral tinnitus

B12. What does tinnitus sound like?

(1) cicada chirping (2) electric current (3) running water

(4) rhythm of the heart (5) others _________

B13. What is the impact of tinnitus on work or life (VAS visual analogue score 0-10)

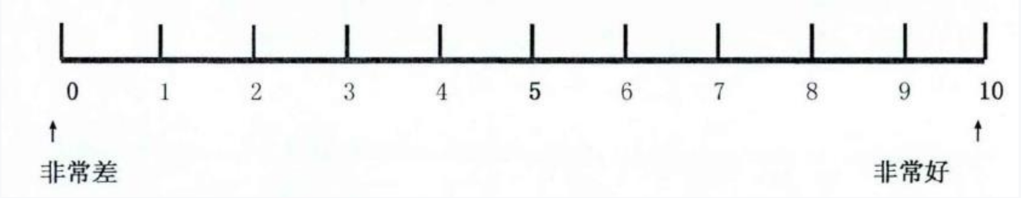

B14. Is there a history of otitis media? (1) Yes. (2) None

B15. Long exposure to noise? (1) Yes. (2) None

B16. Is there a history of ear trauma?(1) Yes. (2) None

B17. Have you used any of the following drugs on a long-term basis (more than two weeks)

(1) Gentamicin (2) Streptomycin (3) Furosemide

(4) Aspirin (5) Erythromycin (6) Cisplatin

B18. Is there any hearing loss in your immediate family member?(1) Yes. (2) No

Physical examination

Note: The subject should rest at least five minutes before the examination.

1.Hight__________ cm ， weight ___________ kg ， BMI___________

2. Waist circumference :__________cm, hip circumference: _________cm

3. Blood pressure: _________ mmHg, heart rate: _________ bpm

4. Thickness of biceps skin ________mm

5. The circumference of upper arm ________cm,

the circumference of upper arm muscle ________ cm

6. Grip strength (left, right) ________

Laboratory examination

1. Serum creatinine ______umol/l，urea nitrogen _______mmol/l，

uric acid _______umol/L

2. Hemoglobin _______g/L, red blood cell _______g /L, serum albumin _______ g/L

3. Blood potassium ________, calcium________, phosphorus________,

PTH________,FGF23_________, Klotho_________

Audiological examination results

1. Otoscientific examination

External auditory canal: ☐ normal， ☐stenosis， ☐congestion， ☐pus

Tympanic membrane: ☐normal ， ☐perforation, ☐hyperemia

2. Pure tone audiometry

	Average hearing threshold	250Hz	500Hz	1000Hz	2000Hz	4000Hz	8000Hz
Left ear							
Right ear							

Investigator: Survey time:

Auditor: Audit time:
