# Supplementary material for: The correlation between fibroblast growth factor-23 and ESRD patients with hearing impairment
Source: PeerJ. 2021 Oct 13;9:e12295. doi: 10.7717/peerj.12295 (PMC8520394; doi:10.7717/peerj.12295)
Supplement: Supplemental Information 3 [file peerj-09-12295-s003.pdf]

代码：□□□□□□□

慢性肾脏病合并听力下降调查问卷

填表说明

A.这份调查表是用来了解您对自己健康状况看法的。这些信息将有助于记录您的感受和您对日常活动的胜任情况。

B.这份调查表包括有关您健康和生活的多方面的问题。

C.请在合适的数字上打个圈,或按要求填写答案。

D.调查表中有一些问题是询问肾脏疾病对您生活的影响,一些问题将会询问肾脏疾病对您生活的限制。有一些问题看起来非常相似,但是每一个问题都是不同的。请认真回答每一个问题。如果您不能确定如何回答一个问题,请尽可能给出一个与您的想法最相近的答案。这将促使我们对不同有肾脏疾病的被访者有更真切地了解。谢谢您参与调查

一、基本情况

A1.姓名：__________，年龄_______，性别：①男　　②女

A2.您读了_______年书

A3. 民族：①汉族 ②少数民族_______

A4. 家庭成员有_____人

A5. 职业①无工作或失业　②农民 ③工人 ④个体户 ⑤职员

⑥离退休人员　⑦干部或教师　⑧其他_______

A6. 婚姻状况：①未婚 ②已婚或同居 ③离婚 ④丧偶 ⑤其他______

A7. 医疗保险：①自费②城镇职工基本医疗保险③城镇居民基本医疗保险④公费医疗

⑤商业医疗保险⑥新型农村合作医疗⑦其他________

A8. 您治疗的医疗机构？______________________（医院名称）

A9. 患病前月收入________元，目前月收入______________元

A10.患病前家庭月总收入________元，目前家庭月总收入________元

A11.您吸烟吗？①不吸（跳至 A14） ②吸，

A12.每天吸________支

A13.吸烟________年

A14. 您饮酒吗？①不饮（跳至 A147） ②饮酒

A15. 每天吸饮酒________两

A16.吸烟________年

A17.您长期服药吗？①不服（跳至 A14） ②服药种类________

二、疾病处理情况

B1. 您的住院诊断是________________________。（住院病历）

B1-1 首次确诊日期：__ _____年_____月

B1-2．您患肾病的病程_____（1）慢性肾脏病一期（2）慢性肾脏病二期

（3）慢性肾脏病三期（4）慢性肾脏病四期（5）慢性肾脏病五期

B2. 您患肾脏病的原发病_____（1）慢性肾小球肾炎（肾病综合征）（2）糖尿病肾病

（3）高血压肾病（4）梗阻性肾病(肾结石等) （5）痛风性肾病（6）多囊肾（7）其他

B3. 有没有合发症？(1)高血压（2）皮肤瘙痒（3）贫血（4）骨痛（肾性骨病）
（5）跛行 (6)腿脚抽筋（7）其他（8）没有

B4. 您肾脏疾病治疗的方式？(1)血透（2）腹透（3）肾移植（4）没有治疗（5）其他_________

B4−1. 开始这种治疗方式的时间：___________年_____月

B5. 自己是否觉得有听力下降？ (1)有 （2）没有 (请跳至 B9)

B6 . 如果自觉有听力下降，听力下降有多久？
（1）数天 （2）半月至 1 月 （3）1 月-6 月 （4）6 月-12 月 （5）12 月以上

B7. 单侧听力下降还是双侧听力下降？ (1)单侧听力下降 (2) 双侧听力下降

B8. 听力下降对工作生活的影响有多大（VAS 视觉模拟评分 0-10 分）

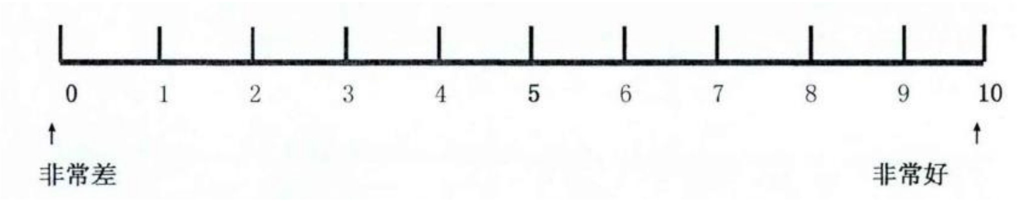

B9. 是否有耳鸣？ (1)有 （2）没有(请跳至 B14)

B10. 如果有耳鸣,耳鸣有多久？
（1）数天（2）半月至 1 月（3）1 月-6 月（4）6 月-12 月（5）12 月以上

B11.是单侧耳鸣还是双侧耳鸣？ (1)单侧耳鸣 (2) 双侧耳鸣

B12.耳鸣声是什么样的？
(1)蝉鸣声（2）电流声（3）水流声（4）和心跳节奏一致（5）其他_________

B13.耳鸣对工作生活的影响有多大（VAS 视觉模拟评分 0-10 分）

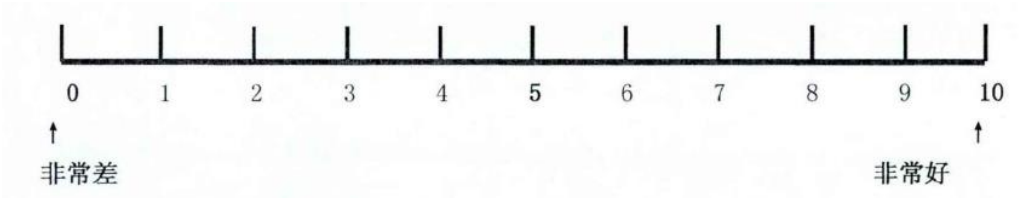

B14. 是否有中耳炎病史？ (1)有 （2）没有

B15. 是否长期接触噪音？ (1)有 （2）没有

B16. 是否有耳外伤病史？ (1)有 （2）没有

B17. 是否长期使用过以下药物（连续使用两周以上）
(1)庆大霉素 (2)链霉素 (3)速尿 (4)阿司匹林 (5)红霉素 (6)顺铂

B18. 直系亲属是否有听力下降的情况？ (1)有（2）没有

体格检查

注意：在检查前，被检查者应该坐着休息五分钟以上。

1. 身高_________________cm，体重_________________kg ，BMI _________________
2. 腰围_________________cm，臀围_________________cm
3. 血压________________mmhg，心率_________________bpm
4. 肱二头肌皮褶厚度________mm
5. 上臂围________cm，上臂肌围________cm
6. 握力（左，右）________

实验室检查

1. 血尿素氮__________,血肌酐_________μmol/L，血尿酸________μmol/L
2. 血红蛋白________g/L，血红细胞________g/L，血清白蛋白________g/L
3. 血钾_________，血钙_______，血磷________，PTH_______，
   FGF23__________，Klotho__________

听力学检查结果

1. 耳科学检查

外耳道：☐正常　☐狭窄　　☐充血/脓液

鼓膜：　☐正常　☐穿孔　　☐充血

2. 纯音测听

	平均听阈（500-4000）	250Hz	500Hz	1000Hz	2000Hz	4000Hz	8000Hz
左耳							
右耳							

调查者：　　　　　　**调查时间：**

审核者：　　　　　　**审核时间：**
